# Challenges and pitfalls when implementing renal replacement therapy in the ICU

**DOI:** 10.1186/cc14727

**Published:** 2015-12-18

**Authors:** Ravindra L Mehta

**Affiliations:** 1 Department of Medicine, Division of Nephrology, University of California, San Diego, 200 West Arbor Drive #8342, San Diego, CA 92103, USA

## Abstract

Several new methods of renal replacement therapy (RRT) are now available for treating patients in the ICU setting. However, utilization of RRT in the ICU is subject to considerable variation and the need for RRT is associated with worse outcomes. Several factors influence the application of dialysis and reflect the interplay of patient and process of care elements that are dynamic in nature. Despite multiple studies evaluating RRT and its application, there are gaps in our knowledge that must be overcome to improve outcomes. This article discusses some of the important issues that require attention in delivering RRT in critically ill patients and provides a framework for the optimal use of RRT in the ICU.

## Introduction

Over the last three decades, various modalities of renal replacement therapy (RRT) have been utilized for managing patients in the ICU setting. Advances in technology have resulted in smarter machines that can be customized to remove solute and manage fluids to meet a wide spectrum of clinical situations. Intermittent hemodialysis (IHD) and peritoneal dialysis (PD), the mainstays of dialysis for end-stage renal disease (ESRD), have been complemented with the development of several modalities of continuous renal replacement therapy (CRRT) [1]. Modifications in operational characteristics and the time available for each session has contributed to newer methods for extended dialysis (e.g., slow low-efficiency daily dialysis (SLEDD)) and have added to the available modalities for RRT [2]. Despite this progress, however, there has been limited success in improving outcomes in patients who are treated with RRT in the ICU. The considerable variation that exists in the application of RRT in the ICU reflects the lack of standardization in this field. The interplay of underlying patient characteristics, process of care, and external events probably contributes to that variation and may influence outcomes. This article reviews important issues to be considered for the optimal use of RRT in the critically ill.

## Challenges for RRT in the ICU

### Determining goals for therapy

Defining the goals of therapy is a key consideration in the use of RRT in the ICU. Critically ill patients span the spectrum of severity at initial evaluation, and changes in severity of illness occur commonly and predict outcomes. Process of care factors (e.g., fluid resuscitation, ventilator requirements, and nutritional support) further influence patient needs. Consequently, therapeutic modalities need to support organ function through the continuum of critical illness. Unfortunately, there is no consensus on the goals for therapy with RRT in the ICU. In most instances, clinicians consider RRT only as a means to address uremic symptoms or biochemical evidence of solute and fluid imbalance. Life-threatening events such as severe hyperkalemia with electrocardiogram (ECG) changes, marked acid-base abnormalities, and diuretic unresponsive fluid overload or central nervous system (CNS) manifestations attributed to uremia are common events managed with RRT. As a result, RRT is only offered when there is clear evidence of functional deterioration to a point at which the kidney is unlikely to recover quickly enough to avoid the deleterious consequences of altered renal function. This approach is largely based on experience with ESRD patients where dialysis is only initiated when the glomerular filtration rate (GFR) has declined to <5-10 ml/minute and the patient is symptomatic. This approach is problematic in ICU patients with acute kidney injury (AKI), because the strategy in treating AKI is to minimize and avoid uremic complications, whereas in ESRD the aim is to keep the patient off dialysis as long as possible. Thus, it is not necessary to wait for progressive uremia as the only goal for RRT. For instance, excessive volume resuscitation, a common strategy used for multiorgan failure (MOF), may result in fluid accumulation and overload even when kidney function is not completely compromised [3]. In these instances, fluid removal to achieve fluid balance could be considered a therapeutic target for RRT. In fact, given the versatility of RRT techniques to maintain metabolic and fluid balance, RRT should be considered a means to maintain homeostasis. CRRT is particularly well suited to provide renal support in the ICU patient. The freedom to provide continuous fluid management permits the application of unlimited nutrition, adjustments in hemodynamic parameters, and achievement of steady-state solute control--benefits which are difficult to attain with intermittent therapies.

### When is the ideal time to start RRT?

Several issues must be considered, including the timing of the intervention, the amount and frequency of dialysis, and the duration of therapy. In practice, these issues are based on individual physician preferences and experience; no set criteria are followed [4]. A major factor is the heterogeneity in presentation of AKI that can occur de novo or can be superimposed on chronic kidney disease (CKD). A lack of specific symptoms other than oliguria further hampers recognition, particularly in ICU patients for whom symptoms that could be attributable to kidney injury may not be as evident and other organs compete for attention. Some observational studies and a few randomized trials have evaluated the timing of dialysis initiation and outcomes [5]. In most of these studies, arbitrary cutoff values of blood urea nitrogen (BUN) and serum creatinine were used to define the timing of initiation as early or late. In critically ill patients with AKI, these biomarkers are not reliable as measures to guide initiation of RRT. BUN levels are influenced by tubular reabsorption of urea, protein intake, catabolism, volume status, upper gastrointestinal bleeding, and use of corticosteroids. Serum creatinine has a nonlinear correlation to kidney function. In AKI, unstable creatinine production, reduced muscle mass, aging, use of drugs interfering with tubular secretion, and variations within the creatinine assay make serum creatinine a nonreliable marker of the GFR. More recent studies have looked at AKI staging criteria and use of novel kidney damage biomarkers to initiate RRT but have had limited success.

Another area of concern that conditions the timing of RRT is the risk for adverse consequences associated with the procedure, including hypotension, bleeding, dialysis catheter-related complications, etc. An "early" RRT initiation would potentially expose a patient who could spontaneously recover kidney function to unnecessary risks [4]. Moreover, RRT could impede kidney recovery and increase the risk of progression to CKD. Whether these risks could outweigh the potential benefits of earlier initiation of RRT is debatable [6-8]. The current lack of consensus forces individual physicians to weigh the fear of adverse consequences against the potential therapeutic benefits [9-11].

Given the uncertainty for identifying an ideal time for initiating RRT, a conceptual framework has been developed that can be applied easily. In our institution, we recommend considering that the kidney has a finite capacity to support other organ functions. Consequently, the need to initiate dialysis should be prompted by the ability of the kidney to meet the demands being placed on it. We propose that RRT could be initiated when there is a mismatch of demand and capacity [7]. As shown in Table 1 several conditions would prompt a mismatch. In most instances, the criteria for initiating RRT would be individualized and based on the existing dynamic conditions rather than on any set of absolute conditions that would need to be met. Although additional research is needed, we would recommend that clinicians consider the decision process of initiating RRT a dynamic one and assess patients for the potential demand and the renal capacity available to meet the demand.

**Table 1:** Renal replacement therapy support based on underlying demand and capacity framework

Demand	Capacity	Example	Action
High	Normal	High catabolic state	Reduce demand if possible
		High nutritional loading	Monitor for renal support
		Poisoning	
High	Low	Decreased GFR from AKI	Renal support
			Reduce demand if possible
Normal	Low	CKD	Add renal support if necessary to maintain steady state
		Noncatabolic	
		AKI	
Low	Low	Malnutrition and wasting CKD	Assess for nutritional state and add renal support if necessary

*AKI *acute kidney injury, *CKD *chronic kidney disease, *GFR *glomerular filtration rate

### What dose should be delivered?

Optimization of the delivered treatment dose has been studied in several clinical trials of dialysis in AKI that have yielded conflicting results [12-15]. These trials have focused on small solute clearances as sole measures of the RRT dose [16]. While solute removal is a key feature of all RRT techniques, operational characteristics differ among delivery modalities (Table 1). Diffusion-based techniques similar to IHD are based on the principle of maintaining a solute gradient between the blood and the dialysate. High blood (250-400 ml/minute) and dialysate (500-800 ml/minute) flow rates maintain the gradient, and the dialysate composition can be varied to achieve solute and acid-base balance. Convective techniques include ultrafiltration and hemofiltration and depend on solute removal by solvent drag [1]. Small molecules are preferentially removed by diffusive methods while larger molecules are more efficiently removed by this convection process; hence, middle molecular clearances are superior. Variations in the time for which RRT is applied (3-4 hours for IHD, 6-12 hours for SLEDD, and 24 hours for CRRT) and alterations in the operational characteristics to harness diffusive and convective clearance distinguish the different modalities. In CRRT the dialysate flow rates are significantly slower than the blood flow rates (typically blood flow rates are 100-200 ml/minute, whereas dialysate flow rates are 1-2 l/hour (17-34 ml/minute)). This flow disparity results in complete saturation of the dialysate [1]. In studies involving CRRT, the dose has been prescribed as a weight-based hourly effluent rate and the delivered dose is considered the measured effluent volume. However, solute clearance may be compromised in delivering the prescribed dose continuously for the full 24 hours due to concentration polarization of the filter, filter clotting, and other factors including access-related problems and external ICU procedures (e.g., surgery, computed tomography scanning) that can reduce total treatment time [17-21]. Similarly in intermittent techniques, hemodynamic instability, shortened dialysis times, and logistic factors often impact adversely on the dose delivered.The effluent rate-based prescribed dose should be incremented by 20-25% to account for decreases in treatment time and lack of filter efficacy in CRRT [24].

Fluid removal to achieve fluid balance is another component of the dose that has not been considered in most trials (Table 2) [22,23]. Fluid removal in RRT is achieved through varying amounts of ultrafiltration that can be tailored to individual need. In contrast to intermittent techniques where fluid balance by necessity depends upon the time available to remove fluid, in CRRT a targeted intervention for fluid balance to achieve any particular hemodynamic parameter is possible [7]. While ultrafiltration requires fluid removal only, hemofiltration necessitates partial or complete replacement of the fluid removed. The composition of the replacement fluid can be varied and the solution can be infused pre filter or post filter. In CRRT, the dose of dialysis delivered is not time dependent. A major distinction for these methods is the ability to dissociate solute removal (e.g., sodium) from fluid balance. As an example, by varying the composition of the replacement fluid or dialysate solute, the balance can be altered and maintained at low, high, or normal levels, while fluid balance can be kept net even, negative, or positive. Fine-tuning of fluid balance on an ongoing basis makes these techniques the most versatile option.

**Table 2:** Proposed parameters for delivered dose assessment

Parameter	Measurement	Tools
**Solute**		
Very small waste products	K^+^, Na^+^, phosphate	Blood levels of K, Na, PO_4_
		Phosphate clearance
	H^-^	pH, HCO_3 _AG, SIDeff, SIDapp, SIG, Delta gap, Delta ratio
Small waste products	Urea	Clearance (ml/minute)
		EKR (ml/minute)
		Standard Kt/V
Middle sized molecules	Serum β_2_-microglobulin	β_2_-Microglobulin clearance
**Fluid**	Weight (kg)	Weight changes
	Inputs-outputs	Fluid accumulation
	Bioelectrical impedance	Fluid overload
	BNP	Bioimpedance vector analysis
		BNP profile

*AG *anion gap, *BNP *brain natriuretic peptide, *EKR *equivalent urea clearance, *H*^- ^ions, *HCO_3 _*bicarbonate, *K *potassium, *Na *sodium, *PO_4 _*phosphate, *SIDapp *apparent strong ion difference, *SIDeff *effective strong ion difference

### What modality should be used?

The primary indication for dialytic intervention can be a major determinant of the therapy chosen because different therapies vary in their efficacy for solute and fluid removal. In the ICU, indications for renal replacement are diverse and require modification based on the clinical situation. For instance, if the indication for dialysis is to facilitate the removal of a drug, such as theophylline in a patient with a drug overdose, IHD is a logical choice, given its efficacy and rapidity of response. If the indication is fluid removal, as in the hemodynamically unstable postcardiac surgery patient, CRRT is preferable. In most patients, however, the indication for dialysis may not be as clear cut, and both solute and fluid removal are desired. In this case, the course of the desired response will also influence this decision. Life-threatening hyperkalemia in an otherwise stable patient is probably better treated with IHD, whereas in the catabolic patient with AKI who is hemodynamically stable the selection would need to be based on other criteria. The presence of MOF can influence the choice of RRT in two ways. First, the presence of MOF may limit the choice of therapies; for example, patients with abdominal surgery may not be suitable for PD because it increases the risk of wound dehiscence and infection [4]. Patients who are hemodynamically unstable may not tolerate IHD[19]. Second, the requirement for anticoagulation depends on the presence of coagulation abnormalities. PD avoids anticoagulation, and IHD can be performed with saline flushes; however, CRRT is difficult without anticoagulation. Recognition of the key features of each of the RRT modalities allows them to be used effectively for the appropriate management of complex patients.

### How long should RRT be continued?

RRT for AKI is based on the premise that kidney function will eventually return and that dialysis can be discontinued. Although this outcome is desirable, it does not always occur. This is particularly true for the patient with AKI and MOF, where the ultimate prognosis depends on the recovery of other organ systems. In this situation, dialytic support may only prolong the time to death and must be instituted only when the goals and endpoint of therapy have been defined. A "trial" of dialysis therapy should be negotiated with the patient's family and other critical care personnel [24]. This trial facilitates withdrawal of dialysis if there is no likelihood of recovery. For instance, an older patient with respiratory, cardiac, and liver failure secondary to sepsis who requires dialytic support for AKI should have a finite period of dialysis (1-2 weeks) and be reassessed for evidence of improvement in all organ systems. If there is no likelihood of recovery, withdrawal of dialysis must be considered.

## Pitfalls for RRT in the ICU

### Maintaining the circuit

A key consideration for all RRT techniques is maintaining the integrity of the circuit to enable adequate solute and fluid management [25,26]. A functioning vascular access that can deliver the required amount of blood flow through the duration of the procedure is a key factor. Patency of the extracorporeal circuit requires the use of anticoagulation which adds to the risk of complications and requires monitoring [27]. Several methods of anticoagulation are now in use and it is possible to minimize the risks. Several protocols are now available for regional citrate anticoagulation for CRRT that have been shown to be more effective than heparin regimens for maintenance of patency [1,28]. Monitoring for anticoagulant efficacy has some unique practical consequences (particularly for CRRT) that often limit effective use of these therapies.

### Achieving fluid balance

Fluid removal is a desirable component of any RRT and is a major goal of RRT in the ICU [22] Volume overload may be an independent contributor to mortality in ICU patients, and thus is important to address, even in the absence of uremia [3]. Fluid removal in IHD is easily achieved in most cases with adequate blood pressure and cardiac performance. However, because the process has to be completed during 3-4 hours every day, the rate of fluid removal has to be high. As a consequence, large shifts in fluid balance generally result and contribute to hemodynamic instability [29]. Additionally, fluid removal--and hence fluid balance--is limited to the period of dialysis. If the patient is hemodynamically unstable during this period it may be difficult to remove any fluid. By contrast, CRRT has the advantage of providing renal replacement continuously and hence fluid removal or replacement can be precisely adjusted for each patient [7]. Because the process is gradual, hemodynamic stability is easily maintained, and these therapies allow ongoing modulation of fluid balance and targeted fluid management. The high efficacy of these therapies in continuous fluid removal lends them for use in situations other than renal failure [22].

### Adjusting drug doses

Drug removal by RRT depends on the molecular weight, charge, and protein binding of the drug [30]. Drugs such as vancomycin and aminoglycosides are easily removed and doses need to be supplemented [14]. Protein-bound drugs (e.g., digoxin) are minimally removed. However, it should be recognized that drug clearances may vary if the circuit is compromised. This is particularly important for CRRT techniques where anticipated drug removal does not occur when the circuit is clotted or the therapy is interrupted [20]. Therapeutic drug monitoring and drug adjustments are necessary to prevent underdosing and overdosing of drugs during RRT. Several resources now exist for managing drug dosing for patients on RRT. The same principles also apply to nutritional support where amino acid losses occur across the filter. Fluid balance can, however, be more easily maintained with RRT, providing an opportunity to maintain adequate nutritional support.

### Procedure-related complications

Complications associated with RRT can result on several fronts. Access-related complications can result from circuit disruptions with subsequent blood loss. Connections should be taped to prevent accidental disconnection. Since CRRT requires anticoagulation for longer periods, the risk for complications related to anticoagulation is higher, although this has not been the case in our experience [1,12,13]. In intermittent therapies, large, rapid alterations in fluid and solutes occur over a short period, so hemodynamic instability--reflected by hypotension and cardiac arrhythmias--is encountered in approximately 25-50% of dialysis patients [31]. Recent studies have demonstrated regional wall motion abnormalities and myocardial stunning in IHD patients that correlate with the volume and rate of fluid removal [32]. A major area of concern is that episodes of hypotension during dialysis can impact negatively on renal and patient outcomes. Most patients treated with CRRT maintain hemodynamic stability and tolerate the procedure well [12,13]. Continuous therapies have the potential for volume depletion, particularly if monitoring is inadequate and calculations are inaccurate. Because large volumes of fluid can be removed quickly, meticulous monitoring is essential, requiring a nurse-patient ratio of at least 1:1, if not more. A major concern is that the continuous nature of these therapies provides a greater opportunity for manipulations by inexperienced personnel. It has been our experience that these can be limited by standardization of protocols for use of these therapies and restricting their use to trained personnel.

### Requirement for mobility

A major consideration in the choice of therapy is the requirement of patient mobility [33]. If patients are to be moved for different investigations, for trips to the operating room, or in the bed for different procedures, continuous therapies become more difficult to implement. Several strategies can be utilized to optimize mobility. Temporary disconnections from CRRT with recirculation through the filter with saline can be done for 2-3 hours to permit patient mobilization. With use of jugular vein access, patients can be moved from bed to chair and can ambulate even when CRRT is running. Structured physiotherapy exercises can be incorporated in the RRT regimen to facilitate patient mobility and return to health.

## Summary

RRT offers a unique set of tools to manage critically ill patients. Although traditional use of RRT has been relegated to patients with severely compromised renal function, they can be applied effectively to provide organ support. Available methods can be selected and adapted to fit any virtually any given situation. Further research is required to identify the patient populations most likely to benefit and to define criteria for appropriate timing of intervention with these therapies. Innovations in technology with newer membranes and combination therapies (e.g., sorbent-based dialysis) are emerging and will enable these therapies to expand to other arenas. It is apparent that to improve outcomes we need to implement our best approaches to optimizing the use of RRT with standardized protocols.

## Abbreviations

AKI, Acute kidney injury; BUN, Blood urea nitrogen; CKD, Chronic kidney disease; CRRT, Continuous renal replacement therapy; ESRD, End-stage renal disease; GFR, Glomerular filtration rate; IHD, Intermittent hemodialysis; MOF, Multiorgan failure; PD, Peritoneal dialysis; RRT, Renal replacement therapy; SLEDD, Slow low-efficiency daily dialysis.

## Competing interests

The author declares that he has no competing interests.

## Notes

This article has been published as part of *Critical Care *Volume 19 Supplement 3, 2015: Future of Critical Care Medicine (FCCM) 2014. The full contents of the supplement are available online at http://www.ccforum.com/supplements/19/S3. Publication of this supplement was supported by Fresenius Kabi.
